# Environmental oestrogens cause predation-induced population decline in a freshwater fish

**DOI:** 10.1098/rsos.181065

**Published:** 2018-10-31

**Authors:** Daniel C. Rearick, Jessica Ward, Paul Venturelli, Heiko Schoenfuss

**Affiliations:** 1 Department of Biology, St. Cloud State University, St. Cloud, MN, USA; 2 Department of Biology, Ball State University, Muncie, IN, USA

**Keywords:** ecological disruptors, predator–prey interactions, endocrine disrupting chemical, population model, behaviour

## Abstract

Understanding population-level effects of environmental stressors on aquatic biota requires knowledge of the direct adverse effects of pollutants on individuals *and* species interactions that relate to survival and reproduction. Here, we connect behavioural assays with survival trials and a modelling approach to quantify changes in antipredator escape performance of a larval freshwater fish following exposure to an environmental oestrogen, and predict changes in population abundance. We quantified the effects of short-term (21 days) exposure to 17β-oestradiol (E2) on the antipredator escape performance of larval fathead minnows (*Pimephales promelas*) and the probability of predation by a natural predator, the bluegill sunfish (*Lepomis macrochirus*). Compared with unexposed minnows, minnows exposed to environmentally relevant concentrations of E2 that approach total oestrogenic activity of wastewater-dominated environments (38 and 103 ng l^−1^) had delayed response times and slower escape speeds, and were more susceptible to predation. Incorporating these data into a stage-structured population model demonstrated that enhanced predation mortality at the larval stage can result in population declines. These results indicate that subtle, sub-lethal shifts in the behaviour of individuals due to human-mediated environmental change can impact species interactions with measurable population-level effects. Such changes have the potential to alter higher-order trophic interactions and disrupt aquatic communities.

## 1. Introduction

Aquatic ecosystems are undergoing rapid environmental change with repercussions for resident wildlife. Human-mediated environmental change, including habitat alteration or loss, the introduction of invasive species and influxes of aquatic contaminants have well-documented adverse effects on the health and viability of aquatic biota [1]. These effects are particularly evident in freshwater species [2,3], which are often geographically restricted and thus especially vulnerable to declines in abundance or extirpation [4]. However, accurately predicting the consequences of environmental change on freshwater aquatic biodiversity remains a challenge; population- and community-level outcomes represent a complex set of dynamic processes that include both effects on species and species interactions [5–8]. Additional and improved studies incorporating species interactions into projections of biological change are therefore a high research priority [9].

Chemical contamination ranks high among the primary threats to the sustainability of aquatic biodiversity. Many aquatic pollutants are oestrogenic endocrine disrupting chemicals (EDCs) entering rivers and lakes via agricultural and industrial run-off and effluents discharged from wastewater treatment plants [10]. These chemicals bind to organismal oestrogen receptors and are potent at very low (ng l^−1^) environmental concentrations. The diversity of oestrogenic EDCs in many wastewater-impacted environments results in aquatic organisms being exposed to a total oestrogenic activity much greater than that of the concentration of any one pollutant. For example, total oestrogenicity (expressed in 17β-oestradiol equivalency values, EEQs) in Boulder Creek, CO, USA has been estimated to be between 31 and 54 ng l^−1^ [11] and up to 50.6 ng l^−1^ EEQ in the Chicago, IL, USA waterways [12]. Oestrogenic EDCs may trigger a cascade of oestrogen-dependent cellular responses that alter the development, behaviour and physiological functioning of aquatic organisms [13–15]. Well-documented molecular, behavioural and physiological effects of EDC exposure on fishes include changes in the growth, behaviour and sexual differentiation of juveniles [16–18], reduced semen quality, inhibited or altered somatic and gonadal growth, altered female fecundity, and changes in male and female sexual behaviour and/or secondary sexual characteristics in adults [19,20]. Exposure to EDCs has also been inferred to be the underlying cause of changes in demographic distributions of natural populations [21], with potentially catastrophic consequences for aquatic biodiversity [2,22].

More recent research indicates that even at environmental concentrations below toxicity thresholds, EDCs have the ability to disrupt ecological processes in freshwater ecosystems [23]. Thus, a central challenge for the conservation of aquatic biota is to understand how contaminant-induced functional deficits within individuals translate into consequences at higher levels of biological organization [7,24,25]. Efforts linking individual-level responses to population-level effects using modelling approaches have demonstrated that changes in the reproductive functioning of adults can directly lead to population declines if physiological impairment of gametes or the timing of reproductive maturation reduce juvenile recruitment below threshold levels required for population persistence [26–32]. Indeed, reproductive failure has been hypothesized to have been a causal factor in the collapse of the fathead minnow (*Pimephales promelas*) population in a lake following chronic exposure to low concentrations of a synthetic oestrogen, 17α-ethynylestradiol (EE2) [2], representing an oestradiol equivalency value of approximately 50 ng l^−1^ EEQ.

A similar level of effort aimed at assessing more complex, behaviourally mediated effects of exposure to contaminants of emerging concern (CEC) on higher levels of biological organization has not been made, despite recognition that an animal's behaviour is an integrated expression of its physiological response to the environment [5] and that human-mediated changes in behaviour can have significant impacts on the viability of populations [33–35]. In part, this deficit is probably because efforts to explicitly link individual-level metrics of exposure to population-level effects using modelling approaches require that the relationships between the endpoints of exposure and survival or reproductive success be known [7,25,36]. Whereas a wealth of compelling evidence exists that demonstrates the adverse effects of EDCs on the behaviour of individuals [5,37,38], few studies have examined how contaminant-induced changes in behaviour translate into changes in fitness [36,39–42].

Here, we connect experimental manipulations with a modelling approach to assess the population-level effects of sub-lethal, EDC-induced behavioural alterations during the early ontogenetic stages of life and show that subtle changes in the behaviour of individuals exposed to CECs have the potential to disrupt ecological processes and influence population trajectories. First, we examined the effect of short-term (21 d) exposure to a natural oestrogen, 17β-oestradiol (E2), on the locomotor mechanics of an innate, evolutionarily conserved, anti-predator escape behaviour [43] using larval fathead minnows (*Pimephales promelas*). Previous work has shown that environmentally relevant concentrations of single oestrogens and oestrogen mixtures alter the evasive performance of larval minnows [17]. However, it is unknown whether such changes translate into an increased susceptibility to predation under more realistic ecological conditions. To address this unknown, we assessed whether exposure to E2 decreased the probability of survival of a mixed group of control and exposed larvae in the presence of a natural predator, bluegill sunfish (*Lepomis macrochirus*). To infer the population-level consequences of sub-lethal changes in behaviour, we imposed the experimentally measured effects of oestrogen exposure on survival in a stochastic, density-dependent, stage-structured population model. Our results help to clarify how anthropogenic environmental change can modify the expressions of simple and complex behaviours within individuals, and the outcomes of interactions between individuals, to affect natural populations.

## 2. Methods

### 2.1. Study subjects, housing and maintenance

Fathead minnow larvae (less than 24 h post-hatch) were shipped at weekly intervals from the US Environmental Protection Agency (EPA) aquaculture facility (Cincinnati, OH, USA) to St. Cloud State University over a six-month period. Adult bluegill sunfish (*L. macrochirus*) were obtained from a certified disease-free aquaculture facility (10 000 Lakes Aquaculture Inc., Osakis, MN, USA). To avoid age and life-history-related biases, the sunfish originated from the same cohort of fish. Throughout the experiment, the study subjects were maintained under ambient summer temperatures and a 16 h light: 8 h dark photo period. Larval minnows were fed twice daily *ad libitum* with newly hatched brine shrimp (*Artemia* spp; Brine Shrimp Direct, Ogden, UT, USA), following established EPA culturing procedures. Sunfish were fed twice daily *ad libitum* with *AquaMax*™ *Grower 400* pellets (Purina Mills, St. Louis, MO, USA).

### 2.2. Water chemistry and quality

The EEQ is a widely used measure of the total oestrogenic activity of an environmental matrix; therefore, E2 can be considered representative of a broad class of chemicals with oestrogenic activity. Reported EEQ values vary substantially across the environment, ranging from a few ng l^−1^ to well over 100 ng l^−1^ [44].

17β-oestradiol (≥98% pure) was obtained from Sigma-Aldrich (St. Louis, MO, USA) and dissolved in 100% ethanol (EtOH) to create a stock solution. The stock E2 and an EtOH solvent control were aliquoted into daily spikes and stored in amber glass bottles at 4°C for the duration of the experiment. Two aqueous exposure solutions (E2_HIGH_ (103 ng l^−1^ E2), E2_LOW_ (38 ng l^−1^ E2)), and a control treatment containing an equivalent volumetric percentage of EtOH were prepared every day via the addition of an appropriate quantity of stock solution to 10 l of conditioned, non-chlorinated well-water. The E2_LOW_ concentration represented total oestrogenicity as reported for wastewater-dominated aquatic environments, while the E2_HIGH_ concentration represented a worst-case environmental scenario (e.g. as could occur after a combined sewer overflow or accidental manure release event [45]). Each solution was thoroughly mixed by agitating the bottles for 10 s and the neck of each bottle was covered tightly with aluminium foil.

Water quality parameters (i.e. dissolved oxygen, total dissolved solids, pH, salinity and temperature) were measured daily using a handheld multi-parameter sampling instrument (model 556 MPS, YSI Instruments, OH, USA). Chlorine was monitored twice weekly using water quality test strips (Hach, CO, USA). In addition, water samples were collected at periodic intervals throughout the exposure period and frozen at −20°C for chemical analysis of E2 via LC-MS/MS (3–4 samples per treatment).

### 2.3. Exposure regime

Larval fish (less than 24 h post-hatch) were exposed to E2_HIGH_ or E2_LOW_, or to the control treatment. Exposure concentrations were within the range of reported environmental values. Approximately 300 larvae were randomly allocated to 1 l glass tanks containing E2_HIGH_, E2_LOW_, or the solvent control (approx. 40 fish per tank). The fish were maintained for 21 days under a 50% daily static renewal protocol, using freshly treated water. The 24 h renewal frequency was well within the environmental half-life of E2 under aerobic conditions (48 h) and prevented the build-up of waste and debris.

Sunfish used in trials were maintained under control conditions (approx. 10 fish/tank) for the duration of the experiment.

### 2.4. Behavioural assays

Two assays were conducted to assess the effect of E2 on predator escape performance and survival. In the first assay, we quantified the locomotor mechanics of escape in exposed and control subjects in response to a simulated (vibrational) predator. In the second assay, we directly compared rates of piscivorous predation among exposure treatments. All trials were conducted on day 22 of the exposure period between 08.00 h and 15.00 h in conditioned well water at room temperature. Each subject was only used once; surviving subjects were sacrificed immediately after each trial via exposure to a lethal concentration of NaCO_2_-buffered MS-222 (Western Chemical, WA, USA).

### 2.5. Escape performance

The behavioural response that we focused on in this study was the ‘C-start.’ The C-start is an evolutionarily conserved, fixed-action pattern response [43], variation in which has been shown to correlate with the probability of surviving a predatory attack [39,46,47]. The response is initiated by the perception of a stimulus and is manifested by bending the body into a C-shape, followed by a burst-swimming bout away from the stimulus. Mauthner cells in the hindbrain drive the escape response by linking the detection of a stimulus to the fixed behavioural response. In turn, a responding central pattern generator promotes motor neuron and interneuron activity, leading to propulsion locomotion generated via muscle contraction. The total response typically occurs within milliseconds [43,48].

**Figure 1.**
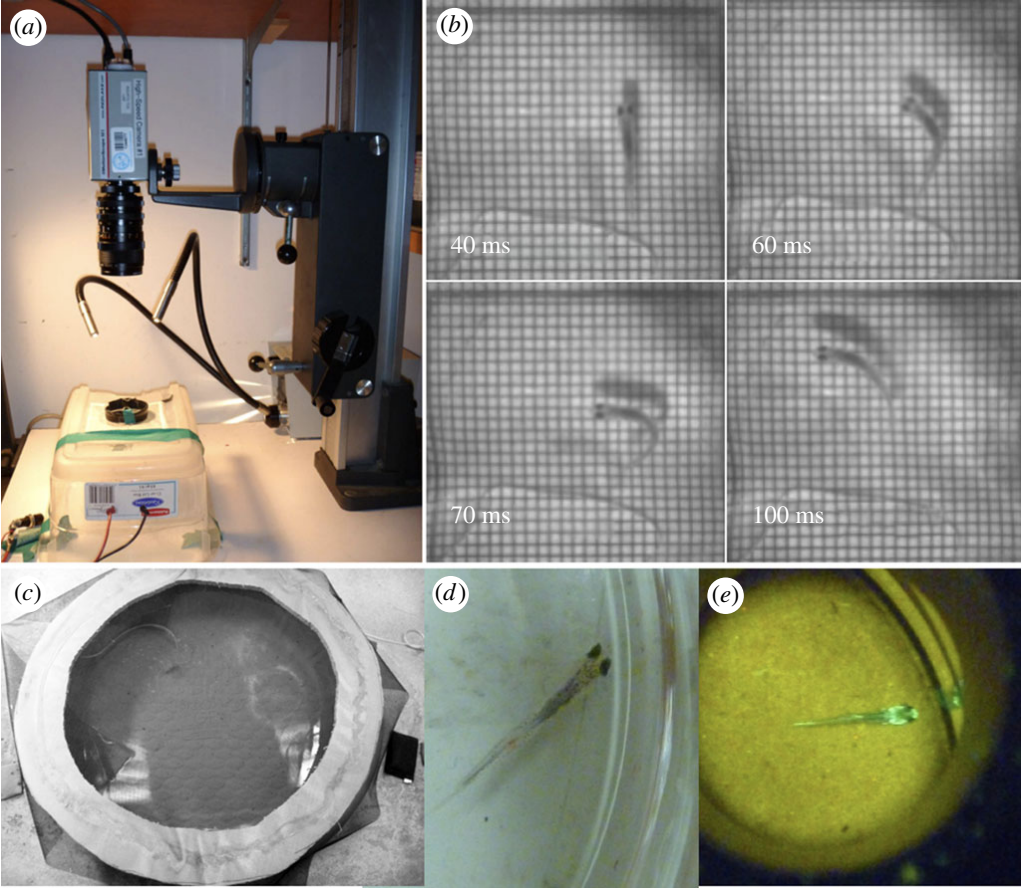
Experimental set-ups for escape performance and predation trials. (*a*) Escape performance was assessed in a testing arena positioned on top of a vibrational chip used to deliver the stimulus and illuminated by a cold-light light source. Trials were filmed using a digital high-speed camera mounted above the arena. (*b*) The fast-start sequence is initiated by a larval fathead minnow after delivery of the vibrational stimulus. (*c*) Photograph of a circular PVC pool used to conduct predation trials. (*d*) A representative unstained larva used in a predation trial. (*e*) A representative SE-MARK calcein-stained larva used in a predation trial, visualized using a fluorescence detector.

We assessed larval escape performance using an established methodology for the quantification of fast-start locomotive mechanics (figure 1*a*,*b*) [17]. Briefly, at the start of each trial, one randomly selected subject was placed into a clear-bottomed, 5 cm diameter testing arena containing 10 ml of conditioned well water. The arena was centred on a pad containing a vibrational chip used to deliver a non-point source stimulus to the subject. The pad was covered with a 1 × 1 mm grid to allow for quantification of the response, and illuminated via a fibre optic light source angled 20 cm above the arena. Subjects were permitted to acclimate to the arena for 1 min, after which the stimulus (approx. 0.5 s in duration) was delivered. Subject responses were recorded using a Redlake MotionScope (Tucson, AZ) high-speed camera (1000 frames s^−1^) positioned approximately 25 cm vertically above the test arena. The objective of this assay was to assess differences in the locomotor response of larval fish. Consequently, only subjects that responded were included in the analysis. If a subject did not respond, it was retested. Subjects were tested up to three times before they were replaced with a new individual. Subjects were tested systematically with respect to treatment (i.e. one control subject, followed by one E2_LOW_ subject and then one E2_HIGH_ subject; *n* = 242–261 per treatment).

We quantified latency to the induction of the escape response, escape velocity, turning angle and total escape response from the videos using the software program *ImageJ* (National Institutes of Health, Bethesda, MD). Two anatomical landmarks, the anterior tip of the snout and the posterior tip of the tail, were digitized on each video and used to calculate standard body length (BL). Two additional landmarks were digitized on the grid at a 1 mm distance to account for scale. Latency was recorded as the length of time to the induction of movement (in ms). Velocity was calculated during the first 40 ms after the initiation of movement, and adjusted to BL per ms. The total escape response of each subject was calculated as BL/latency (ms + 40 ms); we included this parameter because it simultaneously takes into account changes in both velocity and latency.

### 2.6. Predation trials

Trials were conducted in opaque-walled PVC arenas measuring 114 cm in diameter and 15 cm in height, buffered from disturbance by the addition of shade cloth blinds. Each arena contained 83 l of clean, conditioned well water and an airstone (figure 1*c*). Arenas were drained and scrubbed between trials to remove residual chemical cues. Water quality parameters (dissolved oxygen, alkalinity, conductivity and pH) were recorded before each trial using the methods described above.

Up to four trials were conducted simultaneously. To begin a trial, one sunfish (mean total length: 97.76 ± 7.81 mm) was introduced to the arena and given 48 h to acclimate to the set-up. The sunfish was fed 10 non-exposed minnow larvae on both acclimation days to condition it to the food source. At the end of the acclimation period, one group of 10 control larvae and one group of 10 exposed larvae (either E2_LOW_ or E2_HIGH_) were simultaneously introduced to the arena. The sunfish was permitted to feed upon the 20 minnows until approximately 50% of the minnows were no longer visible (trials lasting 2–6 h), as assessed via visual scans of the arena by a single observer every 2 h. Once 50% of the larvae were eaten, the test was stopped and the predator removed via a hand net. Surviving larvae were removed from the arena by glass pipette and transferred to a glass beaker. We calculated the proportion of paired exposed versus control minnows in each trial that survived (*n* = 47 or 53 trials).

To facilitate the identification of survivors in the predation assay, subjects from either the control or the exposed group were marked before the trial using a fluorescent SE-MARK calcein dye (Western Chemical, Ferndale, WA, USA) in accordance with the Investigational New Animal Drug (FDA INAD 10-987) protocol. To prevent mark-associated bias, we randomly selected the treatment to undergo staining in each trial (figure 1*d*,*e*). Subjects to be stained were placed in E2-free, aerated water 12 to 18 h before testing. These subjects were marked through static immersion in the dye for 6 h, at a concentration of 250 mg l^−1^. Pilot trials confirmed that florescence persisted for at least 2 days post-treatment. No mortalities or abnormal behaviours were observed during staining. We counted the number of larvae recovered from each trial using a SE-MARK detector to illuminate fluorescently marked fish.

### 2.7. Statistical analyses

For the predator escape assay, we compared the mean BL of control and exposed (E2_LOW,_ E2_HIGH_) subjects recorded from the escape behaviour assay using a one-way ANOVA, followed by pairwise least significant difference (LSD) *post hoc* tests. Preliminary analysis indicated that the escape performance data were normally distributed (Kolmogorov–Smirnov tests; *p* > 0.05) and satisfied assumptions of linearity. We used one-way ANOVAs to compare the mean response latencies and escape velocities among control and exposed subjects, as well the total escape response.

Because the relative survival of control or exposed fish in any given predation trial was potentially non-independent, we compared the survival of paired control and exposed (either E2_LOW_ or E2_HIGH_) subjects in predation trials using non-parametric Wilcoxon sign rank tests. We compared the survival of larvae from the E2_LOW_ and E2_HIGH_ treatments using a Mann–Whitney *U* test. All analyses were two-tailed and were conducted using SPSS v. 24 (IBM, Armonk, NY, USA).

### 2.8. Population projection modelling

We developed a stochastic, density-dependent, stage-structured population model that tracks eggs and larvae daily, juveniles weekly, and adults annually for 120 years. The model was parametrized using life-history values extracted from well-studied, wild populations in Alberta, Canada [49–52], and modified to include our exposure-associated empirical estimates of larval mortality. Each model year spans 365 days and begins with size-independent spawning on July 25 (the median spawn date reported in [51]). Each female in the model produces eggs that take approximately 7 days to hatch at a mean incubation temperature of 20.9°C [51,52] (table 1). Hatched eggs enter the larval stage and larvae become juveniles after approximately 30 days [51]. Juveniles mature into adults after 1 year [53] and reproduce annually up to a maximum age of 5 years [50]. All individuals in the model are female, assuming a 1 : 1 sex ratio and that sperm is not limiting.

**Table 1.** Population model parameters. All parameters were stochastic and modelled as a triangular distribution with a sample space defined by ±1 s.d. See text for a description of how parameter values were obtained or derived.

parameter (units)	symbol	value (±s.d.)
fecundity (eggs)	*f*	1821 (433)
egg incubation period (days)	*t*_E_	6.93 (0.70)
instantaneous egg mortality rate (day^−1^)	*M*_E_	0.13 (0.04)
larval duration (days)	*t*_L_	30 (7)
instantaneous larval mortality rate (day^−1^)	*M*_L_	0.05 (0.01)
treatment multiplier of *M*_L_	*μ*	1.66 (2.90)
instantaneous juvenile mortality rate (day^−1^)^a^	*M*_J_	0.0130 (0.0008)
adult lengths (age: 1 to 5 years) (cm)	*l*_i_	3.66 (0.38), 5.02 (0.53), 5.67 (0.50), 6.00 (0.41), 6.23 (0.17)

^a^Density dependent; see text for details.

Our model includes life-stage-specific rates of background instantaneous mortality (table 1). Daily mortality rates for eggs and larvae were estimated from [51] and are corroborated by [54]. The model also incorporates daily juvenile and annual adult mortality rates that we estimated by first converting body lengths (2.73 cm for juveniles according to [50,55,56]; and 3.66–6.23 cm for adults following [50]) to mass in g:

$$\mathrm{Weight}=0.0089\cdot \mathrm{Lengt}h^{3.13}$$

[57] and then to mortality rates in natural systems:

$$M=3\cdot \mathrm{Weigh}t^{-0.288}$$

[58]. We repeated this process for 1000 mean juvenile lengths drawn randomly from a normal distribution to estimate the s.d. around mean juvenile mortality. Juvenile mortality is also density-dependent to reflect the competition and predation bottleneck that frequently occurs early in life [59], and to prevent exponential population growth during simulations. Juvenile mortality (*M*) varies positively with juvenile abundance (*N*) according to

$$M^{\prime }=M\cdot (\lambda \cdot N+1),$$

where *λ* is a parameter that defines the sensitivity of *M* to *N*. This equation belongs to a family of Ricker stock-recruitment models in which recruitment decreases at higher densities, such that the resultant curve resembles a positively skewed, normal curve [60]. We calibrated *λ* to 14 777 948 so that the model produced an equilibrium abundance of 2000 adult females (or 4000 adult fatheads assuming a 1 : 1 sex ratio). This is a reasonable population estimate for the 34 ha experimental lake in [2], given the population densities reported by [49].

The model generates 1000 estimates of mean adult abundance over the last 100 years of a simulation for two scenarios: stable conditions in the absence of oestrogen, and a scenario that includes oestrogen effects by incorporating the empirically estimated, 1.66 ± 2.90-fold increase in hourly larval mortality. Fecundity, and non-adult mortalities and stage durations are sampled annually to reflect uncertainty in parameter values as well as environmental variability [49]. To avoid extreme values, these samples are from a triangular distribution with a mode equal to the mean, and upper and lower boundaries equal to 1 s.d. Finally, we used elasticity analysis (±10%) to determine which parameters had the greatest proportional influence on our estimated impact of oestrogen-induced larval mortality on adult fathead minnow abundance. We used R version 3.4.1 [61] for all simulations and associated analyses.

## 3. Results

### 3.1. Exposure parameters and water chemistry

Mean (±s.d.) concentrations of E2 during the exposure period were 38 ± 8 ng l^−1^ (*n* = 3) for E2_LOW_ and 103 ± 17 ng l^−1^ for E2_HIGH_ (*n* = 4). The control treatment did not contain quantifiable concentrations of E2 (*n* = 3; detection limit = 0.1 ng l^−1^). Mean (±s.d.) water quality parameters recorded during the exposure period were as follows: 22 ± 1°C; 5.6 ± 1.3 mg l^−1^ dissolved oxygen; 240 ppm CaCO_3_ alkalinity; 0.83 ± 0.10 mS cm^−1^ conductivity; and 8.4 ± 0.2 pH. Mean (±s.d.) water quality parameters in the predation trial arenas were 21.8 ± 1.24°C; 5.6 ± 1.3 mg l^−1^ dissolved oxygen; 240 ppm CaCO_3_ alkalinity; 0.83 ± 0.10 mS cm^−1^ conductivity; and 8.44 ± 0.23 pH.

### 3.2. Somatic measures

**Figure 2.**
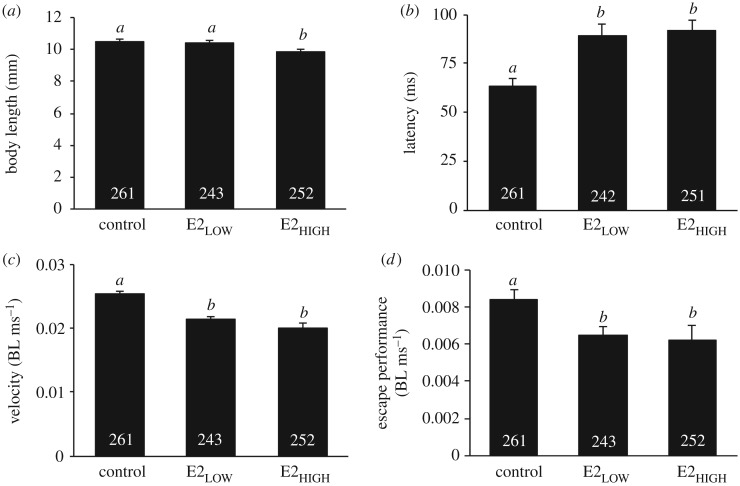
Somatic measurements and escape performance of larval fathead minnows. Variation in somatic indices and anti-predator responses of 22 days post-hatch minnows exposed to 17β-oestradiol (E2) at 38 ng l^−1^ (E2_LOW_) or 103 ng l^−1^ (E2_HIGH_), or to an equivalent volumetric percentage of carrier (control). (*a*) Larvae in the E2_HIGH_ treatment had significantly shorter body lengths (BL, mm) than E2_LOW_ or control subjects. (*b*) Larvae exposed to E2 (E2_LOW_ and E2_HIGH_) had significantly longer escape latencies (ms) than control subjects. The latency of response was recorded from the onset of the stimulus to the initiation of the escape behaviour. (*c*) Larvae exposed to E2 (E2_LOW_ and E2_HIGH_) had significantly slower escape velocities, adjusted for body length (BL ms^−1^), than control subjects. (*d*) Larvae exposed to E2 (E2_LOW_ and E2_HIGH_) had significantly impaired total escape responses (BL ms^−1^), compared to control subjects. Bars and whiskers represent means and standard errors. Sample sizes for each group are indicated within the relevant bar; sample sizes vary due to incompletely recorded performance sequences. For each of the parameters in (*a*–*d*), ANOVAs revealed a significant effect of exposure level (all *p* < 0.001). The letters above each bar reflect the results of pairwise least significant difference (LSD) *post hoc* tests. Groups were considered statistically different if *p* < 0.05.

ANOVA revealed a significant effect of E2 on BL (*F*_2,753_ = 7.42, *p =* 0.001; figure 2*a*). Mean (±s.d.) BLs on day 22 post-hatch were 10.51 ± 2.11, 10.40 ± 1.97 and 9.89 ± 1.75 mm for control, E2_LOW_ and E2_HIGH_ subjects, respectively. *Post hoc* tests (LSD) indicated that E2_HIGH_ subjects were significantly shorter than subjects in either the E2_LOW_ (*p =* 0.003) or control treatments (*p* < 0.001). No difference in BL was observed between control and E2_low_ individuals (*p* = 0.524).

### 3.3. Escape performance

ANOVA revealed a significant effect of E2 on escape latency (*F*_2,751_ = 10.73, *p* < 0.0001; figure 2*b*). Mean (±s.d.) escape latencies for control, E2_LOW_ and E2_HIGH_ subjects were 63.25 ± 65.48, 89.46 ± 84.16 and 91.69 ± 81.65 ms, respectively. *Post hoc* tests (LSD) indicated that subjects from both the E2_LOW_ (*p* < 0.0001) and E2_HIGH_ (*p* < 0.0001) treatments had significantly longer escape latencies than control subjects. There was no difference in the escape latency of subjects in the two E2 treatments (E2_LOW_ versus E2_HIGH_; *p* = 0.749). We also observed a significant effect of E2 on escape velocity (*F*_2,753_ = 7.94, *p* < 0.0001; figure 2*c*). Escape velocities of subjects in the E2_HIGH_ (0.020 ± 0.016 BL ms^−1^; *p* < 0.0001) and E2_LOW_ treatments (0.021 ± 0.015 BL ms^−1^; *p* = 0.006) were significantly slower than those in the control (0.025 ± 0.016 BL ms^−1^). However, subjects exposed to E2_HIGH_ and E2_LOW_ treatments did not differ in escape velocity (*p* = 0.299). We observed a similar effect of E2 on total escape performance (*F*_2,753_ = 3.91; *p* = 0.02; figure 2*d*). Total escape performances of control, E2_LOW_ and E2_HIGH_ larvae were 0.008 ± 0.008, 0.006 ± 0.007 and 0.006 ± 0.012 BL ms^−1^, respectively. The total escape performances of E2_HIGH_ (*p* = 0.01) or E2_LOW_ treatments (*p* = 0.03) were significantly reduced compared to control subjects. Escape performances of E2_HIGH_ and E2_LOW_ subjects did not differ statistically from each other (*p* = 0.750).

### 3.4. Predation trials

**Figure 3.**
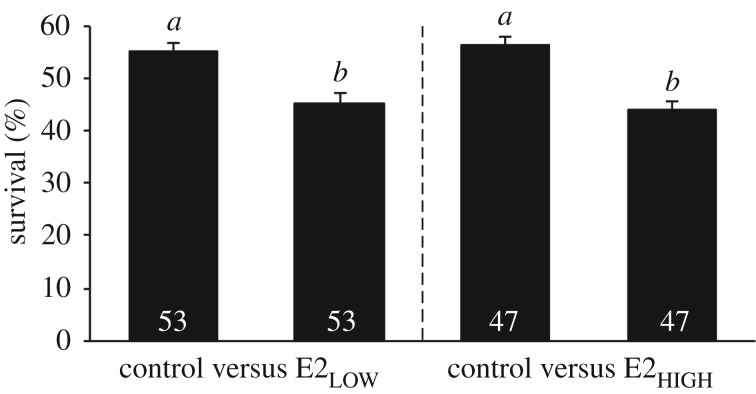
Mortality of larvae under direct predation by bluegill sunfish. The percentage survival of larval fathead minnows exposed to 17β-oestradiol (E2) at 38 ng l^−1^ (E2_LOW_) or 103 ng l^−1^ (E2_HIGH_) was reduced compared with control subjects. Bars and errors represent means and standard errors. Sample sizes (number of trials) are indicated within the relevant bars. The letters above each bar reflect the results of Wilcoxon sign rank tests conducted for paired control and exposed larvae and Mann–Whitney *U* tests conducted between independent groups. Groups were considered statistically different if *p* < 0.05.

The survival of subjects in the E2_LOW_ (Wilcoxon signed rank *Z* = −2.95, *p* = 0.003) and E2_HIGH_ (*Z* = −3.26, *p* = 0.001) treatments was significantly lower than that of paired control subjects (figure 3). In an ecological setting involving a natural predator (bluegill sunfish), the mean (±s.d.) survival of control subjects (pooled), and E2_LOW_ and E2_HIGH_ subjects was 55.4 ± 12.9%, 45.1 ± 13.7% and 43.9 ± 12.0% respectively. The percentage of surviving individuals in the E2_LOW_ and E2_HIGH_ treatments did not differ statistically (Mann–Whitney *U* = 0.457, *p* = 0.648; figure 3).

### 3.5. Population projection models

**Figure 4.**
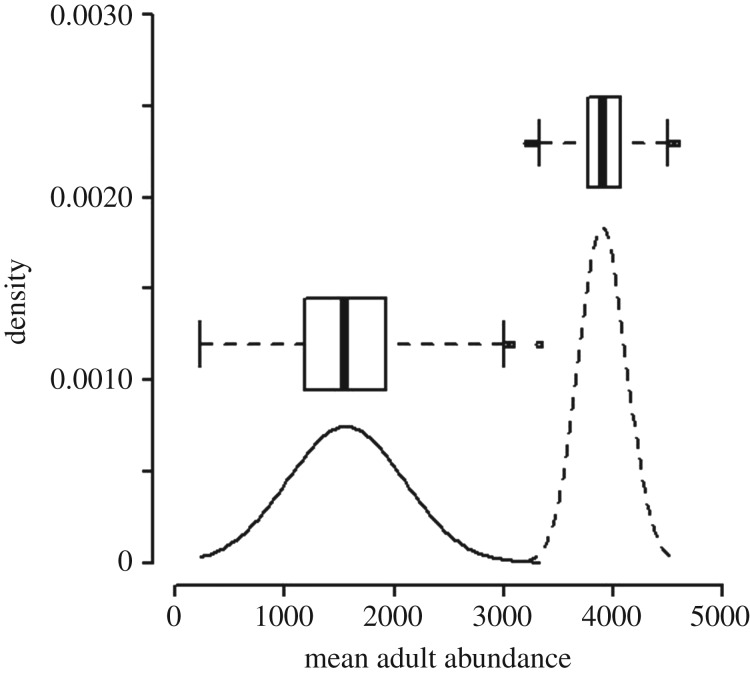
Population-level effects of exposure to 17β-oestradiol (E2). Densities and associated box plots showing the distribution of mean predicted abundance of adult fathead minnows over 100 years for 1000 simulations. Larvae were subject to baseline (dashed line) predation by bluegill sunfish or elevated predation (solid line) as a result of chronic exposure to E2 (mean impact of low and high treatments from figure 3). Density values on the *y*-axis represent the chance that any simulated estimate of mean adult abundance is approximately equal to that value.

A reduction in mean (±s.d.) larval survival from 55.4 ± 12.9% to 44.5 ± 12.85% over two hours translated into a 1.34 ± 0.08-fold increase over the baseline, hourly instantaneous mortality rate estimated from the literature (1.90 × 10^−3^). The model conservatively assumed that the increase in the hourly mortality rate due to oestrogen was present for six hours each day because predatory fishes tend to feed sporadically during daylight [62,63]. The result was a daily instantaneous mortality rate of 5.3 × 10^−2^ ± 3.3 × 10^−2^ for larvae in the presence of oestrogen. This elevated rate of mortality was projected to cause a 60 ± 14% reduction in mean adult abundance in the presence of oestrogen, relative to the pristine condition (figure 4). This result was most sensitive to juvenile and larval mortality rates, and slightly more likely to underestimate rather than overestimate the impact of oestrogen on abundance (electronic supplementary material, figure S1). Larval length, fecundity and the oestrogen-related modifier of larval mortality were also important.

## 4. Discussion

Our study incorporated species interactions into projections of biological change to demonstrate that even subtle, sub-lethal changes in the behaviour of individuals can alter species interactions with marked population-level effects. These data provide important insight into individual-level mechanisms leading to higher-order outcomes of chemical stress on freshwater fish food webs. Predicting the long-term consequences of anthropogenic activity on aquatic biota requires an integrative assessment of the sub-lethal effects of that activity on individuals and the interactions between organisms as they relate to survival and reproduction [7]. Even subtle changes in behavioural interactions that impact fitness (e.g. predator–prey relationships [40,41] or sexual interactions [42]) can alter the structure and function of aquatic communities [5,8]. In this study, exposure to environmentally relevant concentrations of an environmental oestrogen impaired the predator evasion behaviour of larval fathead minnows, and increased the susceptibility of larvae to predation. Incorporating the observed reduction in survival into a population model resulted in a 60 ± 14% decline in equilibrium abundance.

Exposure to E2 altered the escape performance of larval fathead minnows in response to a simulated (vibrational stimulus) predator. Compared to unexposed fish, exposed larvae demonstrated significantly delayed reaction times and reduced swimming velocities. Overall, exposure resulted in a 25% reduction in the total escape performance of larvae compared with unexposed fish. These findings are consistent with the results of previous studies examining the effects of EDCs on larval fishes [64,65], including *P. promelas* [17], that demonstrate that fishes are particularly vulnerable to perturbation by contaminants during the early ontogenetic stages of life. McGee *et al*. [17] examined predator escape in larval fathead minnows exposed to a variety of oestrogens and oestrogen mixes for 5 days as embryos, or 12 days as larvae. They found that a 5-day exposure to 100 ng l^−1^ oestrone (E1) during the embryonic stage was sufficient to delay the onset of the fast-start evasive response in 12-day-old post-hatch minnows. Alvarez *et al*. [39] similarly reported that larval croaker exposed to maternally derived methylmercury (MeHg) as embryos had significantly longer and slower startle responses.

Exposure to EDCs probably affects larval predator evasion behaviour via modulation of the hypothalamus–pituitary–gonadal (HPG) axis, which regulates cellular signalling [66]. Endocrine disrupting chemicals may modulate the HPG axis in two ways that are relevant to the development and expression of an anti-predator response. First, stimulus detection in fish is dependent on a variety of sensory processing mechanisms [43], many of which are regulated by gonadotropin releasing hormone (GnRH). Maruska & Tricas [67] showed that GnRH has an inhibitory effect on neural response to external stimuli in damselfish, suggesting that the perception of a threat stimulus may be altered by EDC-induced modulation of GnRH. Second, impaired escape behaviour may instead be due to modulation of neurotransmitters that affect locomotor abilities [68,69].

Because the behavioural performance of an individual strongly influences the outcome of a predator–prey interaction, exposure to contaminants can be expected to alter the response of an individual in ways that directly impact the probability of surviving a predatory attack [46,70], including variation in predator escape performance [47]. A key feature of this study was that each trial consisted of a mix of exposed and non-exposed individuals, allowing for direct estimates of increased predation mortality due to exposure. In predation trials, the survival of larvae exposed to 38 or 103 ng l^−1^ was reduced by 11% or 10%, respectively, compared to unexposed subjects. Together with findings from the escape performance assays, our data suggest that both a delayed onset of the evasive response and a slower burst-speed swimming speed contributed to this result; Murphy *et al*. [40] found that slower response durations and/or a reduction in the velocity of escape were associated with reduced success in escaping from a predator. More recently, Nair *et al*. [71] reported that enhanced survival during a predatory strike is primarily associated with a greater response distance, suggesting a larger role for earlier sensory detection. Indeed, mortalities associated with exposure have been estimated to be up to 40% greater than those of unexposed larvae, as a direct consequence of these behavioural alterations [40].

Incorporating empirically derived, exposure-induced mortality rates into a stochastic, stage-structured model suggests that altered predator–prey interactions resulting from the sub-lethal behavioural effects of EDC exposure during early ontogeny can lead to population declines. Early-life-stage mortality rates are important regulators of recruitment and year-class strength [72]. Therefore, environmental changes that affect juvenile fitness correlates such as growth or survival [18,73] or alter community-level interactions such as predator–prey relationships or resource competition [74] can impact population growth rate and viability. Moreover, such declines or extirpations of single species can also have persistent indirect effects on aquatic communities. For example, Kidd *et al*. [8] documented a substantial decline in the abundance of predatory lake trout (*Salvelinus namaycush*) following the collapse of forage species in response to EE2, probably due to the reduction in the availability of prey. Concentrations of E2 in the current study matched the total oestrogenic activity of EE2 used in Kidd *et al.* [2] and also matched reported EEQs for urban, wastewater-dominated aquatic environments [11,12]. It is noteworthy that both E2 treatment concentrations affected exposed larval fathead minnows similarly, suggesting a threshold effect at concentrations lower than the 38 ng l^−1^ E2 used here. The prevalence of wastewater-dominated aquatic environments in urban settings suggests that many fish populations are adversely affected by oestrogenic EDC exposure, especially in habitats already degraded by multiple stressors.

Although our results are consistent with the literature, we highlight three caveats to our results. First, a limitation of this study was that predators were not exposed; thus, an assessment of the effects of exposure on the predator is beyond the scope of the study. However, larval fish are often more site-bound than roaming predators [75], and therefore may be more likely to be continuously exposed to pollutant sources. Second, predation trials were conducted in arenas bare of plant life or other prey refugia, which might not accurately reflect the natural habitat of these species. To address these limitations, additional experiments incorporating both predator exposure and refugia into the study design are underway. Such studies examining the interactive effects of exposure on predators and prey under more realistic conditions will better our understanding of how the initial responses of organisms to environmental change translate into changes at higher levels of biological organization and are key to the effective design and implementation of conservation strategies for protecting aquatic biodiversity. Third, we used a relatively simple model to estimate the population-level impact of oestrogen on larval survival in isolation; the actual impact on wild populations will depend on a suit of direct and indirect oestrogenic effects of varying strength occurring within a complex ecological system [76]. For example, our result is conservative if impacts on escape performance extend to other life stages, or if oestrogen reduces male fertility [77,78] or has a relatively small impact on competitors. Conversely, our result is liberal if experimental results do not translate to the field, or if impacts are greater for predators or competitors [79,80]. Estimating population-level effects in the wild will require realistic experiments in which treatments and relevant factors can be monitored or controlled [76]—ideally over multiple generations, and in parallel with population models that more thoroughly incorporate ecological complexity.

We have at present only a limited understanding of how the initial behavioural responses of individual organisms to environmental change translate into effects at higher levels of biological organization. However, as knowledge is gained regarding the mechanisms underpinning shifts in population abundances and distributions, it is apparent that disrupted interactions among species are primary drivers of human-mediated extinction, with declines in food availability being the most common cause [81]. Biotic interactions, including mutualisms, competitive interactions and predator–prey interactions are highly sensitive to the behaviour and physiology of individual species involved and play an important role in the maintenance of biodiversity [82]; this study empirically demonstrates that even highly subtle shifts in the responses of individuals to human-mediated environmental change can drastically alter the outcomes of species interactions, with substantial implications for the sustainability of populations.

## Supplementary Material

Figure S1

## Data accessibility

Raw behavioural data and model code are available from Dryad Digital Repository at: http://dx.doi.org/10.5061/dryad.kk94gp2 [83].
