# Supplementary material for: Environmental oestrogens cause predation-induced population decline in a freshwater fish
Source: R Soc Open Sci. 2018 Oct 31;5(10):181065. doi: 10.1098/rsos.181065 (PMC6227994; doi:10.1098/rsos.181065)
Supplement: Figure S1 [file rsos181065supp1.docx]

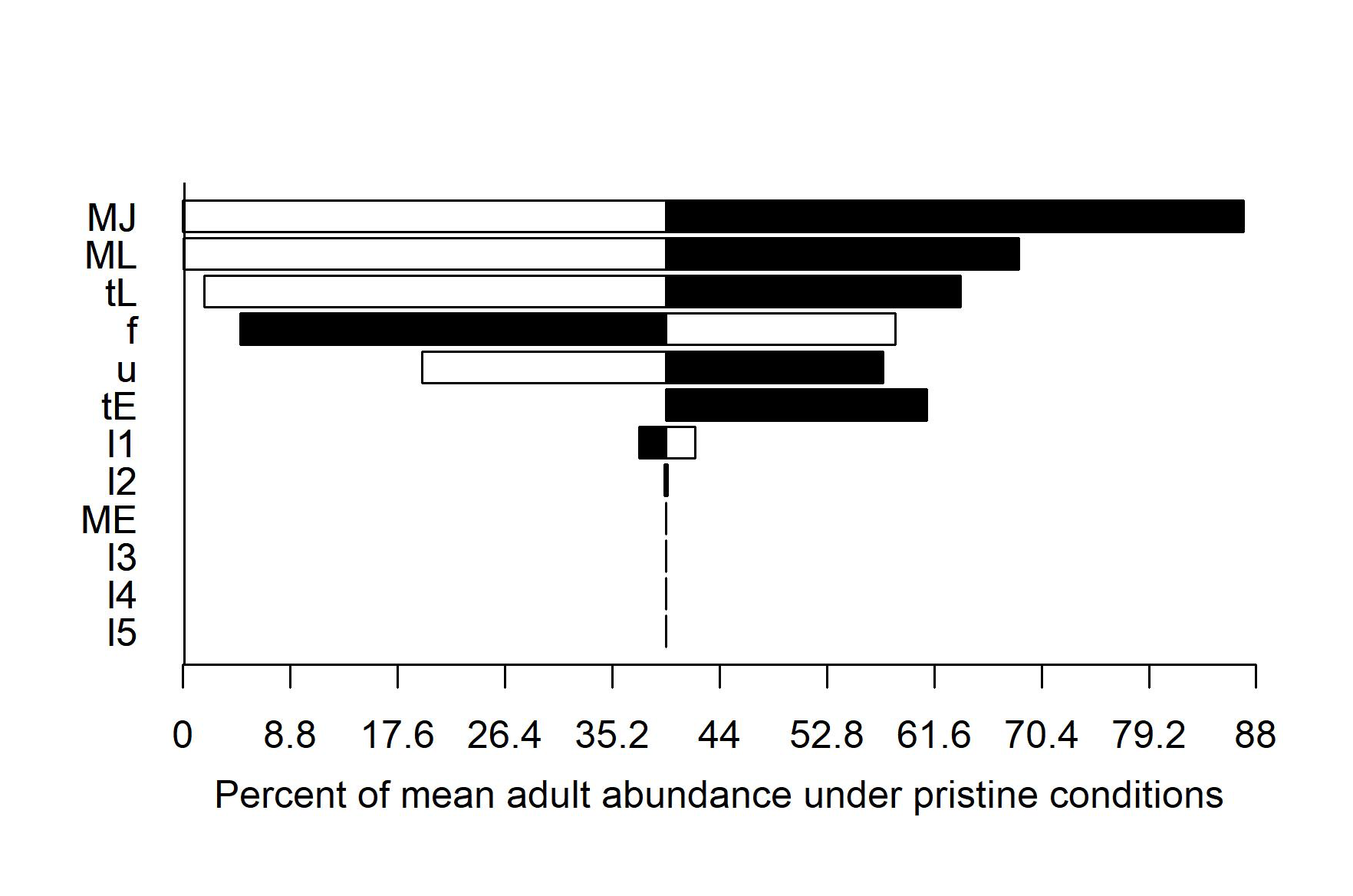


**Figure S1.** Results of elasticity analysis showing the effect of an isolated, 10% increase (open) or decrease (filled) in the mean value of each parameter on the percent of adult fathead minnows that persist when larval survival is impacted by estrogen. The vertical line at 40.1% is the mean estimated impact from fig. 3; bars to the left and right of this line indicate larger and smaller impacts, respectively. Symbols are defined in Table 1.
